# From XMRV to HIV-1 and back

**DOI:** 10.1186/1742-4690-8-S2-O11

**Published:** 2011-10-03

**Authors:** John M Coffin

**Affiliations:** 1 Department of Molecular Biology and Microbiology, Tufts University, Boston, MA 02111, USA

## 

Xenotropic MLV-related retrovirus (XMRV) was first reported about 5 years ago in a few cases of prostate cancer, but did not attract much attention until its reported association with a large fraction of chronic fatigue syndrome cases about 2 years ago. The publication of the XMRV-CFS connection created a ripple of excitement and interest in the scientific, medical, and patient communities reminiscent of the reports of the association of another retrovirus—HIV-with AIDS some 25 years previously. Although I was not directly involved in either discovery at the time, I was a very interested observer who had been working with various retroviruses and who subsequently became involved in both HIV and XMRV research, and I will offer some observations on the parallels and differences between the two. It is noteworthy, that, within 2 years of the first report, in 1983, of the virus we now call HIV, the key findings were widely replicated by virologists using a variety of different assays. By contrast, most of the results of the XMRV paper - isolation of infectious virus from patients, frequent detection of virus in plasma and PBMCs by PCR, detection of antiviral antibodies - remain to be replicated outside of the laboratories that authored the original report despite considerable effort worldwide. HIV is now known as the cause of a global pandemic responsible for large numbers of deaths annually and considerable hardship and economic loss. By contrast, XMRV is considered by most virologists to be the consequence of a collection of artifacts originating from endogenous murine leukemia viruses prevalent in laboratory and wild mice.

In this talk, I will discuss our recent work that uncovered how these associations arose and how even experienced retrovirologists were misled by the initial reports. There are several related, but distinct, issues that need to be considered. First, various mouse (*Mus musculus*) subspecies carry over a hundred different endogenous proviruses closely (>90%) related to XMRV in their DNA. Second, mice are extremely widespread, as is their DNA, which can be found sporadically on laboratory surfaces, as well as contaminants of common reagents and materials. Sensitive PCR assays can detect "XMRV" related sequences in DNA from tiny fractions of one cell. To detect such contamination, we developed a more sensitive assay based on mouse IAP sequences present in thousands of copies per cell. Third, although only a few of the endogenous MLV proviruses encode infectious virus, it has been known since the 1970s that some of them can give rise to virus that can infect human tumor lines when passaged through nude mice. Indeed, A virus identical to XMRV is produced by the 22RV1 prostate cancer line that was derived in just this way In initial reports, however, XMRV did not appear to be sufficiently similar to known proviruses to have been derived this way. However, we have recently shown that this is exactly how it did arise, but not from infection of the precursor CWR22 xenograft with a single virus, but rather with a recombinant between the progeny of two previously undescribed proviruses found in the nude mice used for passage. Since the predicted recombinant is ancestral to all XMRV isolates, and cannot have arisen more than once, it must have found its way into many laboratories as the 22Rv1 cell line was distributed worldwide and, by means that remain to be worked out, into clinical samples from CFS patients.

